# Rectal vs. sublingual misoprostol in cesarean section: Three‑arm, randomized clinical trial

**DOI:** 10.22088/cjim.13.1.84

**Published:** 2022

**Authors:** Fatemeh Zahra Bagheri, Mahboobeh Azadehrah, Bizhan Shabankhani, Ebrahim Nasiri Formi, Hooshang Akbari

**Affiliations:** 1 Department of Operating Room, Mazandaran University of Medical Sciences, Sari, Iran; 2 Department of Gynecologist and Laparoscopy Fellowship, Golestan University of Medical Sciences, Gorgan, Iran; 3 Department of Biostatistics, School of Health, Mazandaran University of Medical Sciences, Sari, Iran; 4 Department of Anesthesiology, School of Allied Medical Sciences, Traditional and Complementary Medicine Research Center, Addiction Institute, Mazandaran university of Medical Sciences, Sari, Iran; 5 Mazandaran University of Medical Sciences, Sari, Iran

## Abstract

**Background:**

Misoprostol is a myometrial stimulant with uterotonic properties and can be administered rectally, vaginally, or sublingually. Numerous studies have investigated the effect of misoprostol on the prevention and treatment of PPH (postpartum hemorrhage) after vaginal delivery, but its use to control PPH during cesarean section has not been widely studied.

**Methods:**

In this clinical trial study, 180 pregnant women who were candidates for cesarean section were included in the study. They were divided into 3 groups of 60 people (sublingual misoprostol group, rectal misoprostol group, control group). In all three groups, the volume of blood lost was recorded in the checklist at the end of surgery. Data were entered into SPSS software and analyzed.

**Results:**

The mean bleeding in the control group was 225.4±63.9, while it was 137.9±33.8 and 118.9±28.5 in the sublingual misoprostol group and rectal misoprostol group, respectively. We had significantly more bleeding in the control group (p<0.001) compared to the other two groups.

**Conclusion:**

These results confirm the positive effect of misoprostol in reducing bleeding and show the superiority of using rectal misoprostol compared to other methods of reducing bleeding during cesarean section.

The most common method of gynecological surgery is cesarean section (CS). Postpartum hemorrhage (PPH) and intraoperative bleeding is one of the main concerns of all obstetricians during cesarean section (1). Different reasons have been offered for cesarean-related bleeding, consisting uterine atony, trauma of the genitals, placental retention in the uterus, surgical incision, insufficient uterine contraction (especially in elective cesarean section), and manual removal of the placenta instead of waiting for separation (2, 3). Bleeding accounts for approximately 25% of maternal mortality worldwide (4). In 70% of cases, uterine atony is responsible for excessive bleeding during or after childbirth (5, 6). Although the surgeon's experience plays an important role in reducing the incidence of primary postpartum hemorrhage in cesarean section, prescribing uterotonics sometimes in large doses helps prevent or stop excessive bleeding from the atonic uterus. These drugs include oxytocin, ergometrine, and prostaglandins (6). Oxytocin is a gold standard uterotonic drug that is usually the first choice in preventing uterine atony in cesarean section; but it has only a half-life of 4-10 minutes (5, 7, 8). Therefore, in cesarean section, oxytocin should be used as a continuous intravenous injection during surgery (5).

Misoprostol is a myometrial stimulant that has uterotonic properties and can be administered rectally, vaginally, or sublingually (7, 9-11). It has minimal side effects such as gastrointestinal symptoms, shivering, pyrexia, fatigue, and headache. Misoprostol is used in obstetrics and gynecology, including abortion, induction of labor, and uterine preparation before surgery(6). Recently, several randomized controlled trials have shown that due to the potent effects of misoprostol on uterine contractions, it is effective in preventing PPH (5, 7). Numerous studies have investigated the effect of misoprostol on the prevention and treatment of PPH after vaginal delivery, but its use to control PPH during cesarean section has not been widely studied (7, 10). Today, misoprostol is used in hospitals in two ways: rectal, sublingual and oxytocin, but due to geographical and ethnic changes, no research has been done in Iran and the better effect of one of these methods has not been proven. Therefore, the present study was performed to preventively compare the reduction of bleeding in cesarean section with misoprostol rectally and sublingually.

## Methods

The present study was a single-blind, randomized controlled clinical trial. The statistical population of the present study was patients who were candidates for elective cesarean section at Shahid Sayad Shirazi Hospital in Gorgan from late July to late September 2020. This research has been registered with the code IRCT20200501047257N1 in the bioethics research site. 

**Figure 1:**
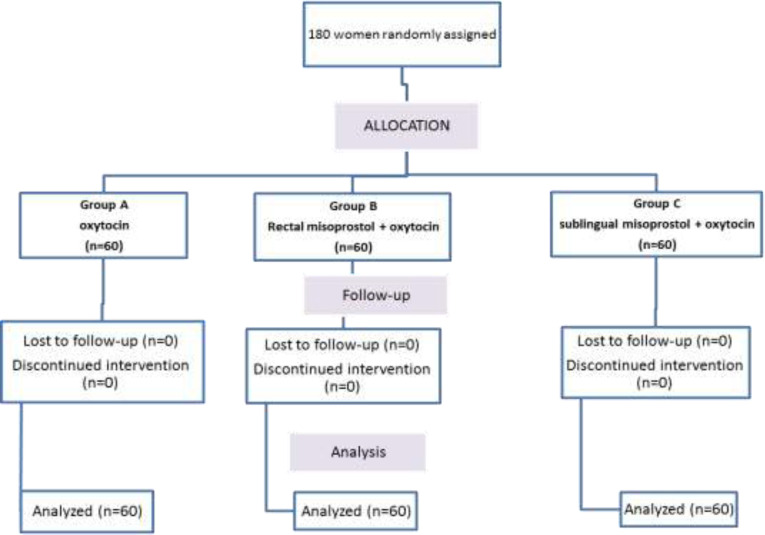
Flow chart of study design

According to the main variable of the study (bleeding during surgery) 180 patients were considered who were divided into three groups of 60 people as A, B and C (group A = sublingual misoprostol + oxytocin, group B = rectal misoprostol + oxytocin, group C or control = oxytocin) (Figure 1). 

Inclusion criteria were: age of patients between 18 and 40 years, candidate for cesarean section, gestational age between 37 and 40 weeks, candidate for spinal anesthesia, written consent of the patient, single pregnancy, and low transverse uterine incision.

Exclusion criteria were: history of PPH, placenta previa and accreta, liver or kidney disease, eclampsia and preeclampsia, epilepsy, height under 155 cm, obesity, infant weight over 4 kg, polyhydramnios (increased amniotic fluid), patients receiving anticoagulants, patients with heart and lung problems and underlying diseases such as diabetes, hypertension (hypertension above 140/90 mmHg), chronic anemia, coagulation disorders, and immunodeficiency. All patients in the three groups were anesthetized with spinal method on the surface of the L3 and L4 vertebrae, with needle number 25 and the drug Marcaine (bupivacaine(*20mg*/*4ml*)) (from MYLAN Co.) before surgery and after the onset of analgesia while they were sitting. After umbilical cord clamping during surgery, the patient received 20 units of oxytocin (10 IU/ml ( (from Alborz Drug Co.) in half a liter of Ringer serum at a rate of 50 cc per minute. Then, the drug was received according to the following protocol:

1. In group A, we placed 200 micrograms of misoprostol (100mcg ((from Abu Reihan Co.) under the patient's tongue.
2. In group B, we placed 200 micrograms of misoprostol in the patient's rectum.
3. In group C or control group, the patients did not receive additional medication.

If there is no proper uterine contraction after a few minutes (maximum 3 minutes), if the initial intervention was not enough, we repeated oxytocin up to a maximum of 100 units (inside Ringer's serum) and if we needed more medication (for groups B and C), we continued misoprostol up to 1000 micrograms. Then the volume of blood lost in each group, at the end of surgery, based on the amount of blood in the suction(12) and also the estimation of the volume of blood absorbed by each number of gauze and long gauze weighted with scales before and after surgery and the volume of clot expelled from the vagina after uterine massage were calculated at the end of the operation and recorded in the checklist (13). To measure the duration of surgery, the time from the moment of incision to the last knot of the skin suture was measured using a timer. Patients receiving misoprostol were evaluated for possible side effects of misoprostol including shivering, pyrexia, nausea, gastrointestinal symptoms and headache. Serum volume and blood intake were recorded in participating patients. The information obtained from each patient was compiled by a researcher-made checklist (consisting of 2 main parts including demographic information and information during surgery). After sampling and data collection, the obtained information was entered in SPSS software and analyzed. 

## Results

180 patients were finally evaluated and the number of patients in all three groups was equal. No patient was excluded from the study according to the inclusion and exclusion criteria. Mean age, gestational age, weight and blood transfusion were the same in the three groups and there was no significant difference in terms of history of cesarean section and race (p>0.05) (table 1). Three variables (duration of surgery, excess fluid, excess oxytocin) were indicated as interfering variables (p<0.05). Therefore, they were examined to observe whether they really acted as interfering variables. Due to the similarity of the slope of regression lines in the three groups for all three variables, it was concluded that none of these variables had an interfering effect during the study. Thus, these three variables had no effect on the results of our study (bleeding). The most common complication in the subjects was nausea, which was 42.4% in group A. The least seen complication in the groups was pyrexia, which showed only 0.01% in group A. Fatigue was the most common complication in group C. Headache was 39.3% in group A, 21.4% in group B, and 39.3% in group C. Also, shivering had the highest percentage in group A and gastrointestinal complications included 43.5% of patients in group B (table 2).

**Table 1:** Information of the studied patients

**P-value**	**Group C** ^3^ **Mean±SD**	**Group B** ^2^ **Mean±SD**	**Group A** ^1^ **Mean±SD**	
0.708	29.3±6.2	28.6±6.6	28.3±6.3	Age(years)
0.986	76.9±12.4	77.1±12.7	77.1±14.4	Weight)kg)
0.328	38.2±1.1	37.8± 0.9	37.7±3.1	Gestational age
0.000	55.2±18.3	49.5±13.7	62.2±14.4	Duration of surgery
0.370	0	0.02±0.13	0	Blood transfusion
0.016	1475.0±666.5	1236.7±469.03	1558.3±664.4	Excess fluid
0.001	34.4 ±21.5	27.6±17.1	41.7±23.1	Excess oxytocin

1. 200 micrograms of misoprostol under the patient's tongue.

2. Placed 200 micrograms of misoprostol in the patient's rectum

3. The patients did not receive additional medication

**Table 2:** Frequency distribution of side effects following the use of drugs in the three groups studied

**EBL** ^1^	**N**	**Mean**	**Std.** ^2^ ** Deviation**	**95% Confidence Interval for Mean**		**P-value**
				**Lower Bound**	**Upper Bound**	
Group A	60	225.4	63.9	208.85	241.92	P<0.001
Group B	60	118.9	28.5	111.62	126.35	
Group C	60	137.9	33.8	129.19	146.65	

1. Eestimated blood loss

2.Standard Deviation

**Figure 2:**
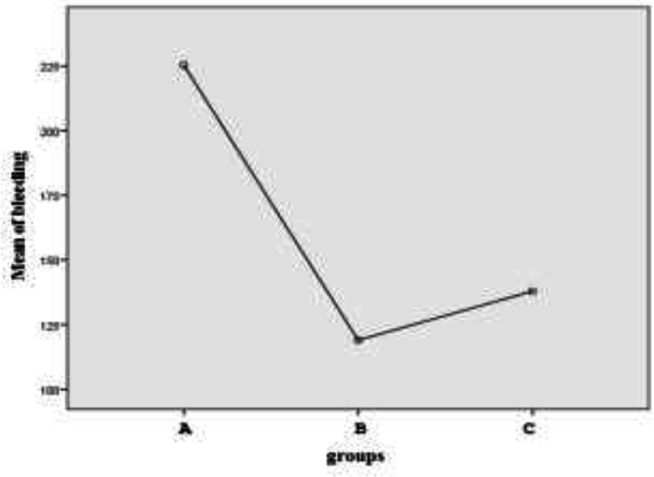
Comparison of mean blood loss in the three groups

As shown in Figure 1, the supplementary tests showed that the mean bleeding was higher in group A compared to the other two groups and we had significantly more bleeding than the other two groups. In fact, the intervention we performed for both groups B and C was effective and the bleeding was significantly reduced. Group B in which rectal misoprostol was used in addition to oxytocin had the lowest mean bleeding and group C in which misoprostol was used sublingually had higher mean bleeding compared to the rectal method (tables 3 and figure 2). 

**Table 3:** Mean and standard deviation of estimated intra-operative blood loss (EBL) in the three groups studied

**Group C** ^3^ **)** **%** **(**	**Group B** ^2^ **(%** **(**	**Group A** ^1^ **(%)**	
32.3	43.5	24.2	Gastrointestinal symptoms
53.5	23.25	23.25	Fatigue
39.3	21.4	39.3	Headache
30	28.3	41.7	shivering
28.3	29.3	42.4	Nausea
0	0	0.01	Fever

1. 200 micrograms of misoprostol under the patient's tongue. 2. Placed 200 micrograms of misoprostol in the patient's rectum. 3. The patients did not receive additional medication

## Discussion

In this study, three groups of rectal, sublingual and oxytocin misoprostol were compared. The results showed that the three groups differed in terms of mean bleeding. The mean bleeding in the rectal misoprostol group was lower than the other groups and was equal to 118.9. The mean bleeding was 138.9 and 225.4 in the sublingual misoprostol group and oxytocin group, respectively. In the study of Eftekhari et al. (7), in line with our study, there was a significant difference in the amount of bleeding so that the mean bleeding in the sublingual misoprostol group (608.9 ml) was less than the oxytocin group (673.9 ml). 

In this study, only patients in the two groups of oxytocin and sublingual misoprostol were compared with each other, but in our study, in addition to these two groups, there was another group that used misoprostol rectally; the least amount of bleeding was reported in the group that used misoprostol rectally, which distinguishes us from the study conducted by Eftekhari. 

The Sitaula (14) and Elsedeek (2) studies also confirm the results obtained in our study, but in these studies only rectal misoprostol was compared with intravenous oxytocin. In another study carried out by Sweed (8) et al., as in our study, individuals were divided into three groups of oxytocin, rectal, and sublingual misoprostol. The results of this study were inconsistent with our study, so that the rate of bleeding in the group receiving misoprostol sublingually was less than the group receiving rectal misoprostol. 

This difference could be due to the fact that in our study, misoprostol was used after umbilical cord clamp, but in the study mentioned, misoprostol was used before the operation. Considering the role of time of drug use on performance and its effect (15), it can be said that the use of misoprostol rectally during surgery has a greater effect on bleeding compared to its use before surgery. On the other hand, in patients who cannot use the drug orally or sublingually, such as people who suffer from nausea and vomiting or patients who have received general anesthesia and are intubated, the rectal method can be considered as a convenient and effective method to prescribe the drug and surgeons and other medical staff are advised to use this method as an effective method to reduce intraoperative bleeding. As other studies, this study had limitations that influenced the quality and the generalization of results. The following limitations can be referred. 

This study was conducted only on women candidates for elective cesarean section, which may limit the generalization of results to other patients with emergency conditions. Another limitation of this study is the time limit for measuring bleeding can be mentioned. More accurate information about the effectiveness and comparison of different methods of misoprostol can be achieved if the amount of bleeding in patients is measured after surgery.

Moreover, in this study, we compared only two methods of using this drug, while there are other prescribed methods such as vaginal and oral, and researchers are advised to compare other methods with these two methods to determine the best and the most effective way to use this drug during surgery to control bleeding. These results confirm the positive effect of misoprostol in reducing the rate of bleeding and show the superiority of this method to other methods of reducing bleeding during cesarean section.

 Finally, the use of misoprostol by rectal and sublingual method is recommended according to the patient's condition and the need to control bleeding during cesarean section, but the dose of the prescribed drug should be careful because misoprostol is a strong drug and its misuse may lead to some risks (16).

## Funding:

The present paper is taken from the master's thesis in field of surgical technology of Mazandaran University of Medical Sciences.

## Conflict of interest:

The authors of the article express that there is no conflict of interest.
